# Contrastive language and vision learning of general fashion concepts

**DOI:** 10.1038/s41598-022-23052-9

**Published:** 2022-11-08

**Authors:** Patrick John Chia, Giuseppe Attanasio, Federico Bianchi, Silvia Terragni, Ana Rita Magalhães, Diogo Goncalves, Ciro Greco, Jacopo Tagliabue

**Affiliations:** 1 Coveo, Montreal, Canada; 2 Bocconi University, Milan, Italy; 3 Stanford University, Stanford, CA USA; 4 Telepathy Labs, Zurich, Switzerland; 5 Farfetch, Porto, Portugal; 6 South Park Commons, New York, USA; 7 University of Milano-Bicocca, Milan, Italy

**Keywords:** Engineering, Electrical and electronic engineering

## Abstract

The steady rise of online shopping goes hand in hand with the development of increasingly complex ML and NLP models. While most use cases are cast as specialized supervised learning problems, we argue that practitioners would greatly benefit from general and transferable representations of products. In *this* work, we build on recent developments in contrastive learning to train *FashionCLIP*, a *CLIP*-like model adapted for the fashion industry. We demonstrate the effectiveness of the representations learned by *FashionCLIP* with extensive tests across a variety of tasks, datasets and generalization probes. We argue that adaptations of large pre-trained models such as CLIP offer new perspectives in terms of scalability and sustainability for certain types of players in the industry. Finally, we detail the costs and environmental impact of training, and release the model weights and code as open source contribution to the community.

## Introduction

### Generalization and scalability in machine learning

The extraordinary growth of online retail—as of 2020, 4 trillion dollars per year^1^—has profoundly impacted the fashion industry, with 1 out of 4 transactions now happening online^2^. The combination of large amounts of data and a variety of use cases has made e-commerce fertile for cutting-edge machine learning (ML) models, with Natural Language Processing (NLP) involved in recommendations^3–5^, information retrieval (IR)^6^, product classification^7^ and many other use cases^8–10^.

However, as the community starts to address the huge operational costs of training and developing models^11^, it is becoming clear that the value of ML innovations has been mostly captured by a few players^12^. Where the rest of the retail industry is making concrete efforts to adapt promptly, companies offering ML products as a service recently gained traction, creating a new multi-billion dollar market^13–16^. The need for ML capabilities that can be applied across entire industries and verticals raises the stakes for an age-old question in ML: *can we build models that can be reused on different tasks and datasets*?

While generalization is a theoretical virtue, real-world models often succeed by (over)fitting to a specific dataset and task^17,18^. In practice, generalization has been considered both hard to achieve *and* economically undesirable for large-scale use cases. In this context, the advent of large-scale, self-supervised models such as *Contrastive Language-Image Pre-training* (CLIP)^17^ is particularly interesting both from a theoretical and a practical point of view. Building upon large pre-trained models to learn *general* concepts in specific verticals/industries (e.g., Fashion, Electronics, DIY, etc.) may provide a new and sustainable way to bring the benefits of ML capabilities to a broader set of practitioners, especially outside of large tech companies. The idea would be to fine-tune general foundational models^19^ to learn concepts that are specific to a domain (e.g., fashion), but general enough to be applicable to all the use cases within that domain.

In *this* work, we show through extensive testing and open-source code that multi-modal training can be successfully used to learn general concepts in a specific domain, namely fashion. In fact, we will argue that it is not only technically possible, but also economically viable, and practically advantageous, since moving away from the traditional setting where single supervised models are trained specifically per use case reduces annotation and maintenance costs while providing solutions transferable across tasks.

### Self-supervised contrastive learning of fashion concepts

Contrastive learning has recently become a predominant approach to learn meaningful representations of concepts in ML. The learning framework builds on the idea that semantically related *concepts* (e.g., two pictures of the same object from different viewpoints) should have *similar* representations, while unrelated ones should be *dissimilar*. Initially devised for self-supervised image representation learning^20,21^, contrastive learning has recently been applied to language as well^22,23^. Recent work has used contrastive training to bridge different modalities, e.g., vision and language^24,25^, audio and language^26,27^, or a combination of the three^28,29^. These models learn concept representations from different modalities (e.g., a textual excerpt such as “a dog running on a field” and a picture depicting the scene) and optimize them to be close in a shared latent space. Crucially, the typical pipeline is self-supervised: since no manual annotation is involved (e.g., in the previous example, one can gather image-text pairs from the web), human intervention is limited to deciding which pre-training task shall be used.

**Figure 1:**
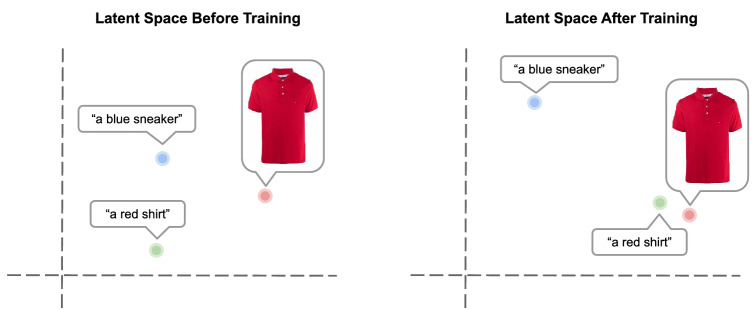
Two-dimensional representation of images and text in FashionCLIP vector space **before** and **after** training. Images and their corresponding textual descriptions are embedded closer to each other in the latent vector space after training.

CLIP^17^ is a vision-language multi-modal neural network trained via CL to associate vision concepts with text. The model comprises a vision and text encoder, each followed by a linear layer to project the image and text representations to the same latent space. CLIP is trained to *position* images and matching descriptions (e.g. an image of a red shirt and its description “a red shirt”) close together in the vector space (see Fig. 1 for an example). When trained on 400 million<image, text> pairs collected from the internet, CLIP has demonstrated competitive zero-shot or few-shot transfer to downstream tasks such as OCR and fine-grained object classification^17^.

More formally, CLIP is a multi-modal model that makes use of an image ($I_{\theta ^I}$) and a text ($T_{\theta ^T}$) encoder. Both encoders are deep neural networks that map raw representations (i.e., an image and a text) to a 512-dimensional dense vector (e.g, given an image *i*, $I_{\theta ^I}(i) \in \mathbb {R}^{512}$). During training, *N* pairs of matching images and texts $<i, t>$ are selected (e.g., as in Fig. 1, the image of a red shirt and the description “a red shirt”), encoded using $I_{\theta ^I}$ and $T_{\theta ^T}$, $L_2$-normalized, and compared pairwise. CLIP minimizes cross-entropy loss such that $\bar{I}_{\theta ^I}(i_j) \cdot \bar{T}_{\theta ^T}(t_k)$ for $j,k = 1,..,N$ is highest when the caption is paired with the correct image ($j=k$), and low otherwise ($j \ne k$), where $\bar{I}_{\theta ^I}(\cdot )$ / $\bar{T}_{\theta ^I}(\cdot )$ are the $L_2$-normalized outputs of the image and text encoders. We summarize the optimization objective for CLIP in Eq. (1) and (2).

1 $$\begin{aligned}&{\mathscr {L}}(\theta ^{I}, \theta ^{T}) = - \frac{1}{2N} \left( \sum _{j} \log \frac{e^{\bar{I}_{\theta ^I}(i_{j}) \cdot \bar{T}_{\theta ^T}(t_{j})}}{\sum _{k^{'}} e^{\bar{I}_{\theta ^{I}}(i_{j}) \cdot \bar{T}_{\theta ^{T}}(t_{k^{'}})}} + \sum _{k} \log \frac{e^{\bar{I}_{\theta ^I}(i_k) \cdot \bar{T}_{\theta ^{T}}(t_{k})}}{\sum _{j^{'}} e^{\bar{I}_{\theta ^{I}}(i_{j^{'}}) \cdot \bar{T}_{\theta ^{T}}(t_{k})}} \right) \end{aligned}$$

2 $$\begin{aligned}&\theta ^{I*}, \theta ^{T*} = {\mathop {\mathrm{arg\,min}}\limits _{\theta ^{I}, \theta ^{T}}}\, {\mathscr {L}}(\theta ^{I},\theta ^{T}) \end{aligned}$$

Here, $\theta ^I$ and $\theta ^T$ are the learnable parameters of the image and text encoder neural networks, and the $(\cdot )$ operator represents the dot product. The first addition operand of Equation 1 is the cross-entropy on the image axis, while the second addition operand is on the text axis.

Recently, industry practitioners have begun to recognize the importance and utility of contrastive pre-training for their target domain, with several works presenting successful downstream applications starting from the CLIP model^30^. In fashion, the multi-modal nature of CLIP has been found helpful in recent discriminative^31,32^ models, which have been developed under the standard paradigm of task-specific, supervised models. In the generative setup, CLIP often complements a larger framework: for example, CLIP is used to learn linguistically grounded codebooks in Variational Auto Encoders^33^ or to guide image synthesis and manipulation in diffusion generative models.^34,35^ While interesting for grounding (see below Fig. 8), the target use case (image generation) and more narrow focus (single task, single dataset) are not readily comparable to FashionCLIP, but instead suggest a possible complementary application in generative use cases. However, no recent CLIP application has been developed to produce industry-wide representations across multiple use cases and datasets. In other words, CLIP has been used only as a pre-trained model, with no attempt to overcome single-task supervised models’ operational and conceptual problems.

In *this* work, we introduce FashionCLIP, a CLIP-based model explicitly trained and tested to produce general product representations for fashion concepts. We train FashionCLIP on a large, high-quality novel fashion dataset: as discussed in the next section, our goal is to establish whether such fine-tuning is sufficient to produce product representations that are transferable in a zero-shot fashion to entirely new datasets.

### Research question and methodology

Standard supervised models for vertical-specific applications such as fashion are costly to train and operate, providing a large barrier to entry for SaaS providers and smaller players^12^. For example, a product classification model might be trained on $<product\,description,\,category>$ pairs derived from catalog data^36^ while optimizing for classification accuracy: if the labels change, or the model is deployed on a different catalog, accuracy would drop. It is important to note that moving to CLIP-based architectures, such as *CMA-CLIP*^37^, does not *ipso facto* solve the problem: if CLIP is used as a per-task model, it will raise the same scalability issues as traditional supervised methods.

**Figure 2:**
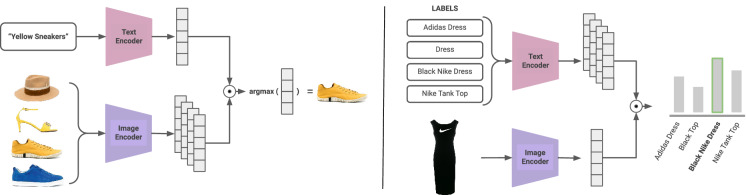
Schematic overview of multi-modal retrieval (left) and zero-shot classification tasks (right).

After training FashionCLIP, we set out to answer a broader, and potentially more impactful question: given the right dataset and fine-tuning procedure, can we learn multi-modal concepts that are general enough for the entire fashion domain? We proceed with a mixture of quantitative benchmarks – inspired both by existing literature and problems known to be important in the industry – and qualitative probes to answer it: since obtaining general concepts is our goal, it is important to verify that FashionCLIP does not only learn a dataset (e.g., an “Armani collection”), but genuine transferable concepts, such as “skirt”, “sleeves”, etc. Taking inspiration from CLIP, our two initial benchmarks will test how FashionCLIP goes from text to image, and vice versa (see Fig. 2):

1. *Text to image* Product search is one of the main channels of interactions and revenues between a shop and its users, accounting on average for 30% to 60% of the total online revenues^38,39^. Historically, product search has been performed chiefly with textual features by first matching queries and product descriptions in an index^40–42^ and then re-ranking the candidate results^43^. However, there are good reasons to believe that including visual features can bring significant improvements since images are often the most curated aspect of the catalog. In contrast, text quality varies throughout verticals, languages, and specific product feeds. Our extensive tests show that FashionCLIP learns fashion concepts, and successfully applies them to unseen products and incomplete or ambiguous descriptions.
2. *Image to text* Product classification is the task of predicting a product category given its meta-data. Classification (even for CLIP-based models, such as CMA-CLIP) is cast as a supervised learning problem where one extracts golden labels from the catalog itself or collects them through crowd-sourcing^7,44^. Generalizing classification to arbitrary labels without constant retraining is again crucial for making ML feasible across numerous players in the fashion industry: transferable concepts help with the interoperability of overlapping, and yet different, fashion taxonomies^45^, a challenge increasingly recognized as central by both practitioners and commentators^46^ (this includes the case of catalogs in less represented languages, for which an English classification is still desirable). Our extensive tests show that FashionCLIP zero-shot capabilities, based on learned associations between vision and textual concepts, allow for quick classification of products in target classes of interest, *irrespective of the specific labeling schemes of individual suppliers*.

We also perform specific probing to understand whether the concepts learned by the model are robust and (somehow) aligned with human semantic intuitions, as opposed to picking up spurious correlations in the dataset^47^:

1. *Grounding*. We probe FashionCLIP for grounding capabilities through localization maps and apply them to the task of zero-shot semantic segmentation for fashion concepts (e.g., sleeve length, texture patterns) and attributes (e.g., *laced* shoes, *glittered* shirts).
2. *Compositionality*. We propose a novel way to probe whether the model can go from semantic segmentation to inferential abilities by composing such concepts to generate new linguistic expressions. We do that through the device of “improbable object”, where a linguistic expression is meant to describe an odd combination of concepts that have never been observed before (e.g., a pair of shoes with handles).

We summarize our contributions as follows:

1. while other researchers have independently developed CLIP-based solutions for individual fashion problems, FashionCLIP is the first explicit attempt to produce general multi-modal concepts for industry: the breadth and nature of our testing methodology make FashionCLIP appealing as a general fashion model, applicable to situations where supervised systems are not practical or viable. Our model is trained on over 700k ${<image,\,text>}$ pairs from the inventory of *Farfetch*, one of the largest fashion luxury retailers in the world, and is shown to be useful in important use cases in a vast global market;
2. we evaluate FashionCLIP in various tasks, showing that fine-tuning helps capture domain-specific concepts and generalizes them in zero-shot scenarios; we supplement quantitative tests with qualitative analyses and offer insights into how concepts grounded in a visual space unlock linguistic generalization. These results would not be possible without the flexibility provided by natural language as a supervision signal and the domain-specific accuracy achieved through fine-tuning;
3. we transparently report training time, costs, and emissions. We additionally release to the community, under an open-source license, training code, a demo app, and plug-and-play checkpoints to help leverage our findings while facilitating ROI considerations^48,49^; the large and unique dataset is also scheduled to be released directly by *Farfetch*. Taken together, FashionCLIP artifacts (model, demo, data) are a foundational toolkit for practitioners in the space and a template for other verticalized CLIP models (https://github.com/patrickjohncyh/fashion-clip).

We believe that our methods and results are interesting not just for the fashion industry but broadly speaking for the ever-expanding industry of online retail, as our artifacts, use cases and benchmarks might serve as a blueprint for other vertical-specific applications of large multi-modal models. Finally, adding to the industry significance of the work, the evaluation in “Grounding and Compositionality” section is new in the context of CLIP-like models, and we believe it may be of independent interest for future work in NLP. As a matter of fact, showcasing a practical thread connecting generalization and latent space interpretation to industrial scalability may be the most interesting contribution of FashionCLIP.

## Results

**Figure 3:**
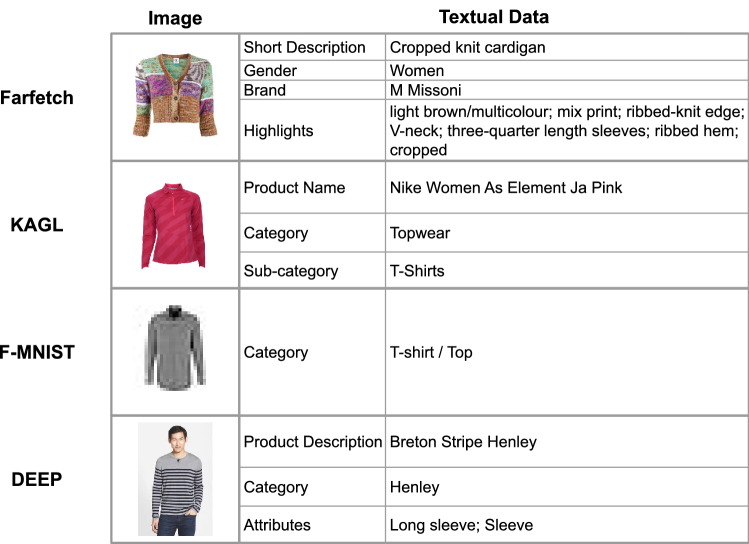
Sample of data from various datasets used. We observe a range of distributions on both the image and textual modalities. For the image modality, we see a range from “Low resolution, B &W” to “High resolution, In-the-Wild”. For the textual modality, Farfetch offers the best “textual resolution”, while DEEP also has very fashion specific terminology. The **KAGL**, **F-MNIST**, and **DEEP** datasets are publicly available. For more details regarding the data, see the Data Availability Section.

In this section, we detail the performance of FashionCLIP over a range of tasks, demonstrating the efficacy of domain adaptation and the applicability of CLIP-like models to fashion. Details on the training and on the evaluation are available in the “Methods” Section. We leverage a variety of in-domain and out of domain datasets, with varying degrees of similarity: **TEST** is the test set from *Farfetch* containing 20k products; **HOUT-C** is the dataset containing a category which we excluded from training; **HOUT-B** is the dataset containing two brands which were excluded from training; **STLE** is a merchandising dataset from *Farfetch*; **KAGL** is a subset of^50^, where each product has a white background image, a caption, and a category; **F-MNIST**^51^ contains 10, 000 gray-scale images from 10 product classes; **DEEP**^52^ contains 4000 product images that are non-standardized (i.e., contain humans) from 50 categories. An overview of image and textual data offered by Farfetch (**TEST, HOUT-C, HOUT-B, STLE**), **KAGL**, **F-MNIST** and **DEEP** can be found in Fig. 3. Our extensive benchmarks and evaluations answer two research questions quantitatively: can domain-specific knowledge improve CLIP understanding of an industry (Fig. 5) and, if yes, does that knowledge translate across different use cases and datasets?

### Multi-modal retrieval

The Multi-modal Retrieval task is described as follows: given a textual description and a set of images, we ask the model to find the image related to that description. For example, a product retrieval task entails matching a product description (e.g., “a red polo for men”) and a photo of it in a catalog.

Multi-modal retrieval is possible due to the optimization objective of FashionCLIP which aligns the language and image latent spaces (see Fig. 1). We test FashionCLIP on multi-modal retrieval to assess the benefits of domain-specific fine-tuning on real-world product search.

Our benchmark takes as input a product description from the catalog’s *test set* and asks models to rank product images corresponding to the caption—the gold standard is the image associated with the product. We extract the ranking using embedding similarities: FashionCLIP performs the dot product between the input caption embedding and each image vector embedding obtained via $\bar{T}_{\theta ^T}(\cdot )$ and $\bar{I}_{\theta ^I}(\cdot )$ respectively and returns a rank based on descending order. We use *HITS@5*^53^ (Hit Rate @ $k=5$) and *MRR*^54^ (Mean Recirpocal Rank) as our metrics. Table 1 compares FashionCLIP against non-domain specific CLIP on different heldout test sets and shows how fine-tuning significantly improves the understanding of our target domain.

**Figure 4:**
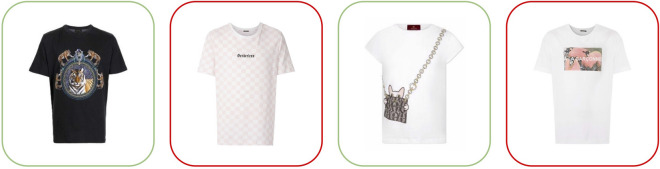
Retrieval with non-fashion concepts. Sample results for “t-shirt with tiger” and “t-shirt with cat” from FashionCLIP (*green*) vs *Farfetch* production search engine (*red*).

**Figure 5:**
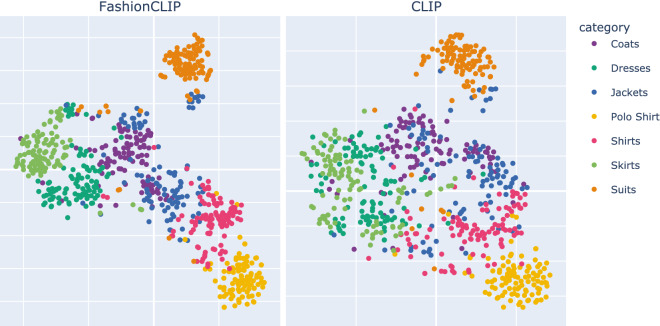
Comparison of F-CLIP and CLIP Image Space T-SNE projection. We observe better clustering (0.115 vs 0.0745 silhouette score^58^) in F-CLIP for categories such as Shirts, Skirts and Dresses, where the products form a denser cluster with less overlap between categories, suggesting that the F-CLIP latent space is better tuned for fashion concepts.

We also perform extensive qualitative tests comparing FashionCLIP with the production search engine presently employed in the catalog. Fig. 4 shows a case of particular interest for product search: in this example, visual concepts do not belong to the fashion domain and are not available in the caption. The first comparison (*left*) shows that FashionCLIP can recover the concept of *tiger* when prompted with “t-shirt with tiger”; for the same query, the search engine retrieves items matching the category, unable to interpret *tiger* based solely on text. The second comparison (*right*) shows that FashionCLIP can interpret *a cat* from a stylized, partially occluded drawing. In contrast, the search engine fails to generalize beyond the captions explicitly containing the string “cat”. Finally, visualizing the learnt embeddings (Fig. 5) also helps to build an intuition of FashionCLIP’s better conceptual resolution when it comes to the target domain.

**Table 1:** Comparing FashionCLIP (F-CLIP) vs CLIP on the multi-modal retrieval task.

Model	Dataset	HITS@5	MRR
F-CLIP	TEST	**0.66**	**0.50**
CLIP		0.28	0.21
F-CLIP	HOUT-C	**0.62**	**0.47**
CLIP		0.33	0.23
F-CLIP	HOUT-B	**0.58**	**0.41**
CLIP		0.31	0.22

Best performing models are in bold.

**Table 2:** Comparing the performance of FashionCLIP (F-CLIP) on product classification task over several datasets (**F1** is *weighted macro F1*).

Model	Dataset	F1
F-CLIP	TEST	**0.39**
CLIP		0.31
F-CLIP	KAGL	**0.67**
CLIP		0.63
F-CLIP	F-MNIST	**0.71**
CLIP		0.66
F-CLIP	DEEP	**0.47**
CLIP		0.45

Best performing models are in bold.

### Zero-shot classification

We replicate CLIP’s original zero-shot classification setup^17^, which allows us to quantitatively assess the transferability of FashionCLIP’s fine-tuned representations to different data distributions from the same vertical (i.e. Fashion). The model generates *one* image embedding for the product image, and *k* text embeddings, one for each of the labels in the classification scheme (e.g., “shoes”, “shirt”). The predicted label is the one that is closer (measured via dot product) to the image in the model’s vector space. We use *weighted macro F1*^55^ as the performance metric. Table 2 summarizes the results of different SOTA benchmarks. On all the tested benchmarks, FashionCLIP is superior to CLIP, a result which suggests that domain-specific fine-tuning is indeed useful in-domain and that it generalizes to other, completely unseen datasets.

Furthermore, we set out to investigate the “cheating hypothesis” on our domain-specific model, i.e., the hypothesis that supervised models do not generalize as well as CLIP because they fit spurious features unique to each dataset. We freeze the image encoder from FashionCLIP and fine-tune a linear classifier, LINEAR, over the embeddings generated on a subset of categories (47) from the validation set from *Farfetch*. We run benchmarks on $\mathbf {TEST_S}$, $\mathbf {KAGL_S}$, $\mathbf {F}$**-**$\mathbf {MNIST_S}$ and $\mathbf {DEEP_S}$, sub-sampled versions of the respective datasets. Where labels are different, we adapt LINEAR to the labels by pooling the scores from relevant classes. We compare this to zero-shot performance, using the original labels to generate the text embeddings.

Table 3 reports our findings, which are partially similar to those from CLIP^17^. Given that **F-MNIST** is very different from **TEST**—comparable, for example, to CIFAR-100^56^ vs. ImageNet^57^—the decrease in performance may be an indication of cheating. However, LINEAR performs well on the other datasets, with the biggest gain for **KAGL**, whose product image most resembles those in **TEST** (i.e., high-resolution items on a white background). Compared to the original setting^17^, one may argue that the supervised model has an easier job in our case: much fewer categories ($$10^1$$ vs. $$10^3)$$ and relatively homogeneous items, **F-MNIST** aside.

**Table 3:** LINEAR classification performance relative to zero-shot on F-CLIP (**F1** is *weighted macro F1*).

Dataset	F-CLIP	LINEAR	$\Delta$F1
$\texttt {TEST}_\texttt {S}$	0.746	0.900	+ 0.154
$\texttt {KAGL}_\texttt {S}$	0.764	0.881	+ 0.117
$\texttt {DEEP}_\texttt {S}$	0.411	0.444	+ 0.033
$\texttt {F}-\texttt {MNIST}_\texttt {S}$	0.781	0.602	− 0.179

**Table 4:** F1 macro on **STLE**; Prior classifies using empirical class probabilities.

Model	Man	Woman
Prior	0.24	0.20
F-CLIP	**0.36**	**0.27**
CLIP	0.33	0.17

Best performing models are in bold.

While we leave the investigation of fashion classification in more ecological settings as future work, our results contain actionable insights for real-world deployments. In particular, supervised classifiers still require a good deal of manual intervention even for similar datasets, and they are utterly unusable on neighboring yet different problems. Table 4 reports performance on **STLE** divided by Man- and Woman-related items. Products in the dataset still come from *Farfetch*, but labels are manually assigned by merchandisers and are orthogonal to the taxonomy (*classic, streetwear, edgy* vs. *shoes, hats, bags*). The versatility afforded by language supervision allows zero-shot models to tackle the challenge by simple prompt engineering (“an item in *classic* style”); in contrast, supervised models would require a new training and evaluation pipeline. As emphasized above, learning general fashion concepts is the main motivation behind *this* work: while specific, supervised pipelines may still be the best choice for specific problems, they are no longer the only viable option in multi-tasks scenarios thanks to the advent of large-scale models such as FashionCLIP. Although no single answer can fit all the use cases, we wish to encourage data-driven decision-making by charting all the options and providing cost and performance assessments.

### Grounding and compositionality

As argued in the *Introduction*, given that we are interested in establishing a connection between generality and scalability through large multi-modal models, it is important to further evaluate the quality of the learned representations. While the question of whether FashionCLIP *learns* fashion has been addressed quantitatively above, we are also interested in evaluating the model from a broader theoretical perspective of language understanding, offering a glimpse of the extent of FashionCLIP’s “true” generalization capabilities, *ala* “infinite use of finite means”^59^.

The literature on language compositionality spans centuries: limiting ourselves only to recent work, *grounding* has been explored in connection with efficient learning^60,61^, and “true understanding”^62,63^. Using combinatorial principles to test generalization abilities is a known strategy in toy world^64,65^: we exploit insights from our target domain to operationalize similar principles on *real-world* objects.

In this section, we provide evidence of semantic grounding in FashionCLIP and build on that to offer a preliminary investigation of its compositional abilities. Our analysis starts from two lessons from previous research. First, *localization maps*^66,67^ are an effective way to probe the model for *referential* knowledge^68^ (we borrow here the referential/inferential distinction from the classic work by Marconi ^69^) and visually grounded lexical knowledge. Second, from a linguistic point of view most search queries in fashion have the form of Noun Phrases (NPs)—e.g. “armani dress”. Therefore, the semantics of NP can be considered a good real-world generalization^9,70^ for studying FashionCLIP *compositional* and *inferential* abilities.

**Figure 6:**
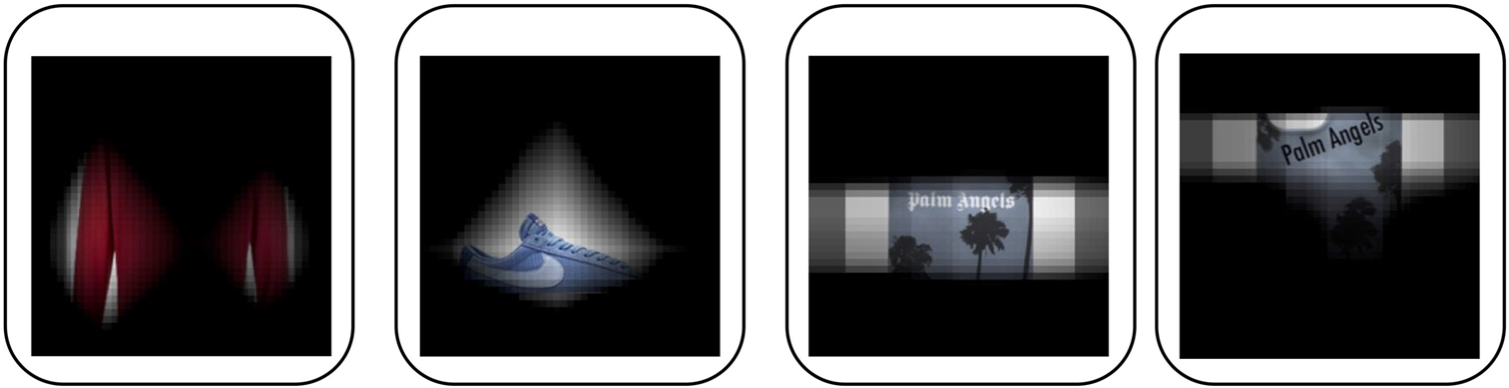
Grounded lexical knowledge. Maps are easy-to-use probes into the model fashion knowledge. *Left to right*: localization map for “long sleeves” on a red polo; sneakers and the map for “Nike”, a phone cover and the map for “Palm Angels”; the same phone cover and map, when the logo is written with an out-of-distribution font in a new spot.

#### Grounding

We probe FashionCLIP for evidence of referential knowledge and investigate its grounding capabilities by utilizing localization maps. We further apply localization maps to the task of zero-shot fashion parsing—a crucial open problem in the industry ^71^.

Localization maps are obtained by repeatedly occluding different parts of the image. We then encode each occluded version and measure its distance from the target text in the contrastive space. Intuitively, the farther the image is pushed away by the occlusion, the stronger the linkage was between the removed visual concept and the text and, in turn, the higher its score on the map. Fashion parsing is a specific case of semantic segmentation where bounding box annotations contain clothing items. We extract bounding box annotations (as an approximation of fine-grained segmentation) from localization maps by finding the minimum bounding rectangle of highly activated areas.

**Figure 7:**
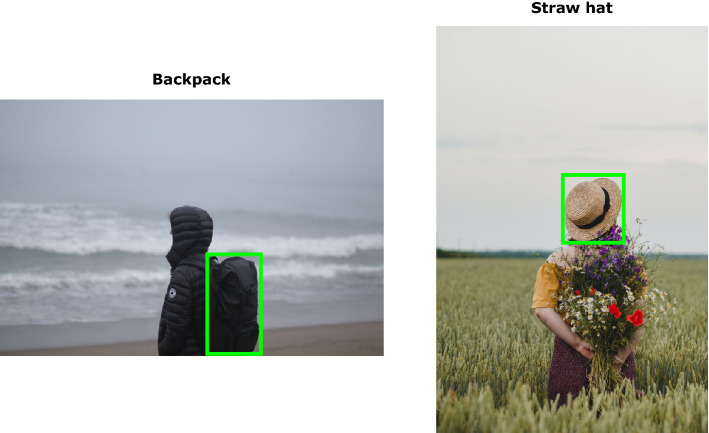
Item bounding-box detection. Localization maps can be easily extended to provide zero-shot bounding boxes for items of interest.Green bounding boxes show the predicted locations for fashion concepts “Backpack” (left) and “Straw hat” (right). Images above are taken from the publicly available Unsplash Lite Dataset 1.2.0: FashionCLIP was tested extensively on ModaNet - please reach out to authors for links to those images.

**Figure 8:**
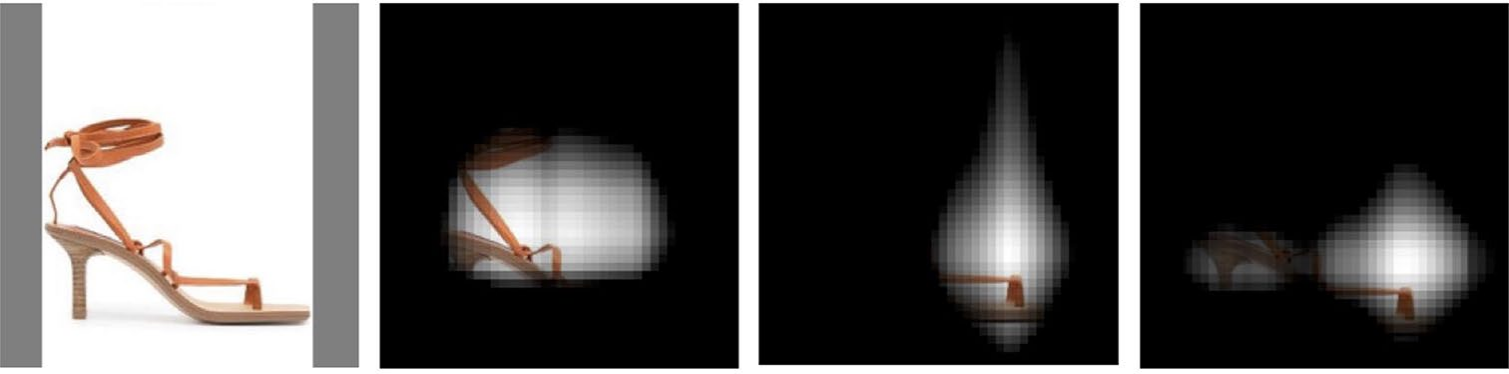
Grounding and compositionality. Localization maps for a product retrieved with the query “ankle strap sandals with high heels”: left-to-right, the product, “ankle strap”, “sandals”, “high heels”).

As shown in Figs. 6 and 8, features such as “high heels”, “ankle strap”, “long sleeves” are well represented in FashionCLIP; the model also seems to be very aware of brands, in more or less explicit form. FashionCLIP picks up the abstract logo on *sneakers* (Fig. 6), as well as showing (similar to CLIP) good OCR capabilities, when recognizing a logo as an explicit text string. Fig. 7 shows zero-shot bounding box annotations of some samples in the previously unseen ModaNet^72^ dataset. While it is unlikely that zero-shot models could replace specialized segmentation training, we believe that models such as FashionCLIP could provide a cheap way to generate probabilistic labels for weak supervision pipelines.

#### Compositionality

Given the preliminary evidence that isolated concepts reliably map onto visual regions, our working hypothesis is that FashionCLIP should exhibit true *inferential* abilities by *composing* such concepts to generate new NPs.

**Figure 9:**
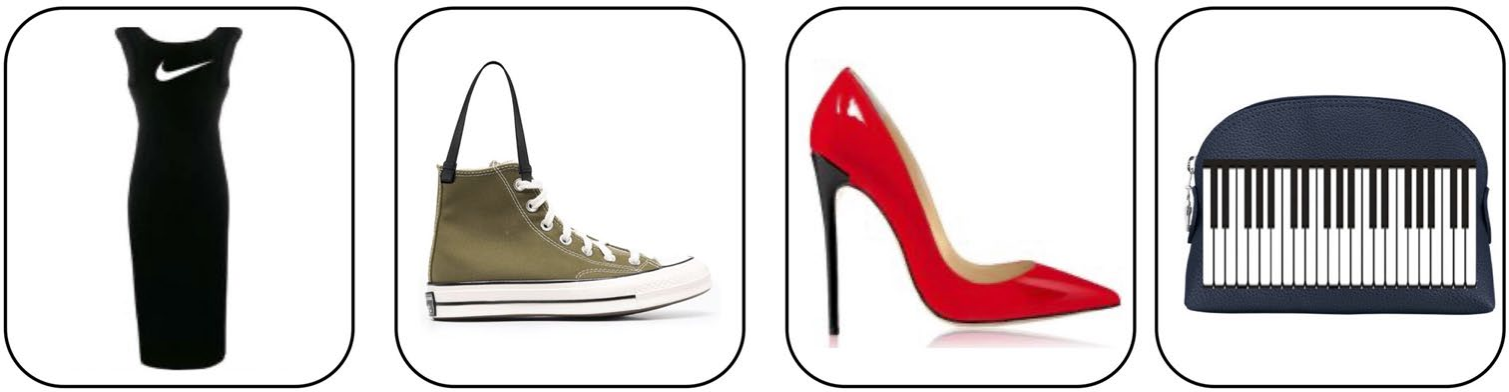
Improbable products. By combining fashion features, brands, and items in new ways, we obtain visually realistic products with clear, zero-shot compositional semantics. From left to right: “Nike long dress”, “converse with handles”, “red shoes with black high heel”, “keyboard pochette”.

We build on domain knowledge, previous literature^52^ and *Farfetch*’s inventory to probe the model for knowledge of *brands* (e.g. “nike”), *features* (“high heels”), and *drawings* (“keyboard”), manually verifying the text-to-region mapping for each of these concepts via localization maps. Given that these single concepts are grounded in regions (Fig. 8), we could leverage this knowledge to generate new images and NPs *systematically*. Crucially, we can assign a defined semantics to a new *brand + object* NP that describes an “improbable object” that has never been seen before (Fig. 9). Improbable objects vary: they may portray odd combinations of concepts, such as a *Nike long dress*, a surreal item, *sneakers with handles*, or an unlikely extension of existing fashion items, such as the *keyboard pochette* (which generalizes the theme first found in J. Mugatu’s *keyboard tie*). A new NP such as “nike dress” would require the visual region corresponding to the word *dress* to contain the visual region of the logo corresponding to the word *nike*.

We supplement our analysis by re-purposing our classification and retrieval pipeline: in the classification task, FashionCLIP achieves an accuracy of 0.74 when asked to pick the improbable label out of a set of credible distractors. The following are examples of test cases:

- *target*: *NIKE DRESS* (as seen in Fig. 9), *labels*: Nike dress, an Armani dress, a shirt, the flag of Italy, a Gucci dress, a Nike t-shirt;
- *target*: *BLACK SHOES WITH RED HEEL*, *labels*: black shoes with red heel, black shoes, red shoes with red heel, red shoes with black heel, red shoes, fuchsia shoes, the flag of Italy, sneakers, black sneakers, a bag.
- *target*: *RED SHOES WITH BLACK HEEL* (as seen in Fig. 9), *labels*: black shoes with red heel, black shoes, red shoes with red heel, red shoes with black heel, red shoes, fuchsia shoes, the flag of Italy, sneakers, black sneakers, a bag.

For the retrieval task, we add the new images to **TEST**, and use the NPs as queries: out of 20k products, the model’s top choice is correct half the time (*HITS@1* $$ =0.53$$), a percentage that quickly rises to 0.82 with $$k=5$$ (as a comparison, CLIP scored *HITS@1* $$=0.51$$ and *HITS@5* $$=0.73$$).

While a full-fledged investigation of compositional abilities is beyond the scope of *this* contribution, FashionCLIP inferences on improbable products suggest the presence of *some* degree of compositionality: important fashion concepts are “identifiable” in the latent space and can be singled out and re-combined into unseen concepts, exhibiting on a small scale the creative generalization we usually associate with symbolic systems^73^. In addition, the ability to distinguish “red shoes with black heel” from “black shoes with red heel” implies knowledge beyond a bag-of-words semantics^74^.

Recent research suggests that CLIP’s compositional capabilities are limited.^75^. As shown by our results, restricted domains allow for direct manipulation, without the risk of confounding; indeed, restricted domains may be easier to explore but further investigation is needed to confirm compositional capabilities. Furthermore, as suggested by the use of the MASKClip objective introduced in the *ARMANI* model^33^, adding explicit visual segmentation may induce better discrimination for certain fashion concepts. While more costly losses are an interesting area at the intersection of grounding and compositionality, given both the narrow generative focus and the magnitude of the improvements in the original paper^33^, their conclusions cannot be readily applied to FashionCLIP. We look forward to performing future research combining insights from generative and discriminative use cases.

## Discussion

FashionCLIP is a domain-adaptation of CLIP, motivated by central use cases in fashion^71^: differently from *task-specific supervised* methods, FashionCLIP does not need a specialized architecture, labeling, and tuning. We extensively verified the flexibility afforded by language supervision, and investigated FashionCLIP’s semantic capabilities on new tasks. Our focus on a specific industry allows not just practical gains but also opens up theoretical possibilities by constraining the domain, which is still large, but also easy to manipulate. By providing quantitative and qualitative evidence that contrastive learning, coupled with a large and diverse dataset, can indeed produce general multi-model industry concepts, we connect theoretical virtues with significant practical gains, and open new possibilities for scaling the horizontal deployment of machine learning systems in an effective way.

As a truly general system, FashionCLIP concepts could be used for many more tasks: for example, multi-modal representations can be features in downstream systems, or directly used for zero-shot recommendations in item-to-item scenarios^76^; classification over arbitrary labels could be used as a fast and scalable labeling mechanism, supporting probabilistic labeling^77^ or data generation for multi-modal IR models^78^. While leaving this (and many other themes) to future iterations, we do believe *this* work—with its artifacts and methodology—to be a first, rounded assessment of the great potential of general, transferable, multi-modal concepts for digital commerce.

**Figure 10:**
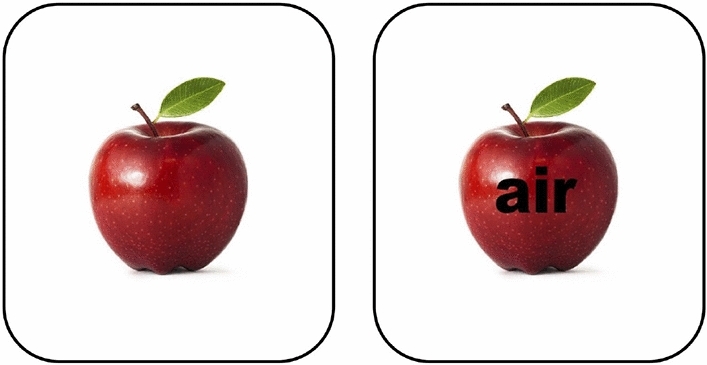
Typographical attack. FashionCLIP correctly identifies the object to the left as an “apple”, but misclassifies the one to the right as “nike air”, as the text acts as a confounder^79^.

The authors are aware of the risks of multi-modal *CLIP*-like models in production associated with their limited robustness, as well as general issues with biases in large language models pre-trained at scale. In particular, we acknowledge that the risk of adversarial attacks on multi-modal models is an area of active research^80,81^. To the limits of our knowledge, we have no reason to believe that FashionCLIP introduces any *additional* risk when compared to the original CLIP. As with the original model, it should be noted that FashionCLIP appears to be susceptible to “typographical attacks” (Fig. 10). No datasets used for training or testing contain PII and/or other sensitive user data.

## Methods

### Training dataset

*Farfetch* made available for the first time an English dataset comprising over 800 k fashion products, with more than 3k brands across dozens of object types. Compared to other large fashion datasets, our dataset is significantly more complete than DeepFashion^52^, which lacks detailed text descriptions, and even larger than CM-Fashion^33^, which has been collected without any direct involvement by *Farfetch*. Items are organized in hierarchical trees, producing a three-layer taxonomy: for example, *trees* could be something like *Clothing> Dresses> Day Dresses* or *Clothing> Coats> Parkas*, for a total of 800+ trees. As input for the image encoder, we use the standard product image, which is a picture of the item over a white background, with no humans (images follow a specific set of rules regarding the placement of the item, lights of the photo, etc., designed to highlight the item’s features); as for the text, *Farfetch* has two types of text, *highlight* (e.g., “stripes”, “long sleeves”, “Armani”) and a *short description* (“80s styled t-shirt”). See Fig. 3 for an example.

We create a training, validation, and test set from the catalog by random sampling products. Our final training and validation sets comprise 700 k and 50 k products respectively from 188 categories.

### Training pipeline

We apply fine-tuning starting from the pre-trained CLIP with the following parameters: we use Adam Optimizer with betas in (0.9, 0.98), epsilon of 1e−6 and weight decay equal to 0.2 and three different learning rates [1e−4, 1e−5, 1e−6]. We train the models for 4 epochs, evaluate every 500 steps and select the model with the lowest validation loss for each configuration (Table 5, model selected in bold). In our preliminary tests, the model with the lowest validation loss overall did not generalize the best in the zero-shot setting. This poses an interesting question, left for future work, of how to fine-tune these large pre-trained models without losing in generalization. The pipeline has been implemented with Metaflow^82^, with training executed remotely on cloud GPUs; experiment tracking was provided by Comet^83^.

### Testing datasets

We prepare the following datasets for testing purposes and to further gauge the potential impact of the model in production at scale. **TEST** is the test set from *Farfetch* containing 20k products; **HOUT-C** is the dataset containing a category which we excluded from training (*Performance Tops*), for a total of 1.5k items; **HOUT-B** is the dataset containing two brands which were excluded from training, for a total of 1.7k items; **STLE** is a merchandising dataset from *Farfetch*, completely independent from the catalog, that classifies 7749 items across 6 styles for gender women and 4 styles for gender men; example of styles are *Classic* and *Streetwear* and each item may belong to more than one style; **KAGL** is a subset of^50^, where each product has a white background image, a caption, and a category, for a total of 9990 items over 62 categories; **F-MNIST**^51^ contains 10, 000 gray-scale images from 10 product classes, with pixel intensity inverted to obtain images with white background (note that these images have a size of 24 × 24 thus showing much less details than the images on which the models have been trained on). **DEEP**^52^ contains 4000 product images that are non-standardized (i.e contains humans) from 50 categories.

### Training FashionCLIP

We re-purpose the CLIP main architecture^17^, which we describe briefly in the *Introduction* for the sake of completeness. In the end, we obtain a multi-modal space where images and texts are jointly projected and learned: if training has been successful, we expect that, for example, the textual embedding for the string “red long dress” is actually similar (as measured by the dot product) to the image embeddings of red dresses. Table 5 shows training time, performance, and costs.

**Table 5:** Comparing training time, performance, costs, and carbon emission on variants of the FashionCLIP architecture on the *Farfetch* catalog.

LR	Loss	Time(m)	USD	kgCO_2_ eq
1e−4	16.0	618	31$	0.77
1e−5	1.73	617	31$	0.77
**1e−6**	**2.83**	**621**	**31$**	**0.78**

Cost is calculated with the AWS pricing for a *p3.2xlarge*; estimations were conducted using the Machine Learning Impact calculator^84^. Model used for testing in bold.

## Data Availability

The **KAGL**, **F-MNIST**, and **DEEP** datasets are publicly available. The Farfetch dataset is scheduled to be released in the near future. As part of the ongoing mission to help the retail space leverage the latest A.I. techniques and to promote multidisciplinary research in data science across industries, Farfetch is working to finalize the release of the dataset used in this study under a research-friendly license. Please check https://github.com/Farfetch for updates on the data release, and reach out to the authors for preliminary inquiries.
