# Publisher Correction: Contrastive language and vision learning of general fashion concepts

**DOI:** 10.1038/s41598-022-26364-y

**Published:** 2023-01-23

**Authors:** Patrick John Chia, Giuseppe Attanasio, Federico Bianchi, Silvia Terragni, Ana Rita Magalhães, Diogo Goncalves, Ciro Greco, Jacopo Tagliabue

**Affiliations:** 1 Coveo, Montreal, Canada; 2 Bocconi University, Milan, Italy; 3 Stanford University, Stanford, CA USA; 4 Telepathy Labs, Zurich, Switzerland; 5 Farfetch, Porto, Portugal; 6 South Park Commons, New York, USA; 7 University of Milano-Bicocca, Milan, Italy

Correction to: *Scientific Reports* https://doi.org/10.1038/s41598-022-23052-9, published online 08 November 2022

The original version of this Article contained errors in Figures 2 and 7 where the images did not display correctly. The original Figures 2 and 7 and accompanying legends appear below.

**Figure 2:**
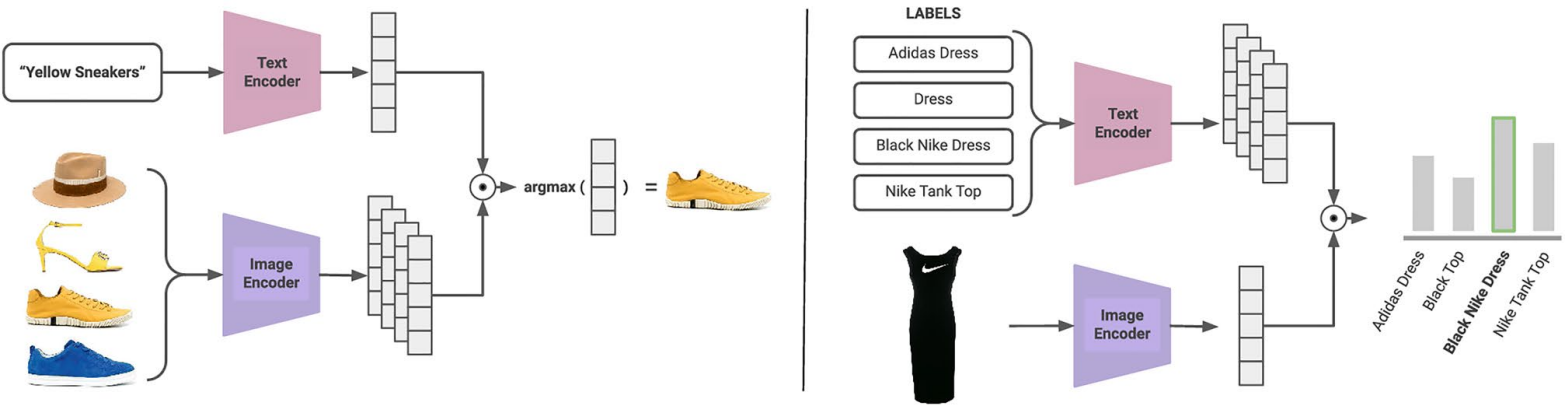
Schematic overview of multi-modal retrieval (left) and zero-shot classification tasks (right).

**Figure 7:**
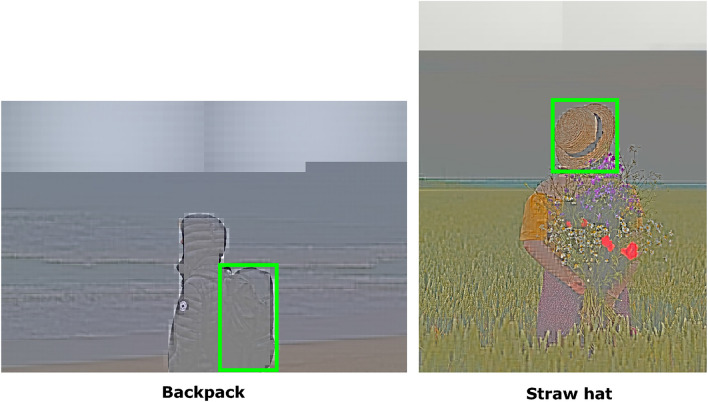
Item bounding-box detection. Localization maps can be easily extended to provide zero-shot bounding boxes for items of interest. Green bounding boxes show the predicted locations for fashion concepts “Backpack” (left) and “Straw hat” (right). Images above are taken from the publicly available Unsplash Lite Dataset 1.2.0: FashionCLIP was tested extensively on ModaNet - please reach out to authors for links to those images.

The original Article has been corrected.

